# Exploring a Flow Cytometry-Based CFU Assay for Functional Assessment of Human HSPCs: a Robust Alternative to Morphological Colony Evaluation

**DOI:** 10.1007/s12015-025-11045-w

**Published:** 2025-12-26

**Authors:** Anne Louise S. Revenfeld, Anaïs M. J. Møller, Mette Tylvad, Marie Bill, Carina A. Rosenberg, Bjarne K. Møller

**Affiliations:** 1 Center for Gene and Cellular Therapy, Department of Clinical Immunology, Aarhus University Hospital, Aarhus, Denmark; 2 Department of Hematology, Aarhus University Hospital, Aarhus, Denmark

**Keywords:** Hematopoietic stem and progenitor cells, Differentiation, Colony-forming unit assay, Flow cytometry

## Abstract

**Background:**

Assessment of hematopoietic stem and progenitor cells (HSPC) function is essential for both research and clinical applications. Traditional colony-forming unit (CFU) assays, based on morphological evaluation in semi-solid media, are labor-intensive and subject to operator bias, limiting reproducibility and standardization.

**Methods:**

We evaluated a commercially available, flow cytometry-based liquid CFU assay for quantifying HSPC proliferation and differentiation. Using a high-throughput platform, colonies were identified and classified by the expression of CD14, CD15, and CD235a (Glycophorin A). Results were compared with conventional semi-solid CFU assays.

**Results:**

The flow-based assay demonstrated strong correlation with traditional methods, while reducing both hands-on time and inter-operator variability. The approach allowed objective, reproducible classification of BFU-E, CFU-G, CFU-GM, CFU-M, and CFU-GEMM colonies, and was compatible with clinically relevant sample types.

**Conclusion:**

The liquid CFU assay provides a standardized, efficient, and robust alternative for functional evaluation of human HSPCs, supporting its broad adoption in both research and clinical settings.

**Supplementary Information:**

The online version contains supplementary material available at 10.1007/s12015-025-11045-w.

## Introduction

 The proliferation and differentiation capacity of hematopoietic stem and progenitor cells (HSPCs) can be evaluated ex vivo, using colony forming unit (CFU) assays. These assays represent a valuable tool for researchers and clinicians in their effort to understand the biology, function, and therapeutic potential of HSPCs in a wide array of applications [1]. The CFU assays aim to quantify the number of multipotent progenitor cells, by formation of single clone colonies, as well as to identify the specific colony types, consequently allowing assessment of the self-renewal capacity and lineage potential of HSPCs, respectively [2].

The most widely used CFU assays are based on seeding HSPCs in semi-solid methylcellulose-based medium, containing a mixture of factors to support proliferation and differentiation of HSPCs into multi-lineage hematopoietic prodigy. The evaluation of the resulting colonies is performed by using light microscopy and classification of colonies based on their morphological features [3]. Scoring and counting of colonies is a laborious and subjective procedure, which can be substantially affected by operator experience, consequently introducing a high degree of variability [4–6]. This particularly challenges the standardization requirements of assays used in a clinical or cell manufacturing context [2, 7].

To address this, efforts have been made to automate scoring, or to refine or supplement the classical semi-solid CFU assay with other alternative assays, including single-cell RNA sequencing [8] and immunofluorescent staining of colonies directly in the semi-solid medium [3, 4]. As an alternative approach, flow cytometry-based CFU assays offer a promising solution, minimizing operator bias and hands-on time while leveraging widely adopted analytical platforms in haematology and immunology. Here, we evaluate a novel, commercially available liquid CFU assay that utilizes antibody staining and flow cytometry for colony identification. The assay employs a 96-well, methylcellulose-free format compatible with high-throughput flow cytometric analysis. Like the traditional CFU assay, cells are evaluated 14 days post-plating, allowing for colony formation and proliferation. Colony numbers and types are assessed by the relative expression of a combination of three different surface markers; CD14, CD15, and CD235a (Glycophorin A). Based on the distribution of the marker expression, the colonies are assigned as burst-forming unit-erythroid (BFU-E), colony-forming unit-granulocyte (CFU-G), colony-forming unit-granulocyte-macrophage (CFU-GM), colony-forming unit-macrophage (CFU-M), or colony-forming unit-granulocyte-erythroid-macrophage-megakaryocyte (CFU-GEMM). The performance of the flow-based CFU assay was compared to a traditional semi-solid CFU assay and the assay applicability is evaluated with clinically relevant samples. We demonstrate that the liquid CFU assay is a reproducible and robust assay in combination with standard flow cytometry and data analysis platforms, requiring relatively few gating strategy modifications between experiments and thereby minimizing data analysis time. The most critical points are the combination of surface markers and the limits for colony assignment, which calls for careful evaluation and possible adjustments depending on the sample type - such as leukapheresis product, isolated CD34 + cells, or culture expanded cells - and the specific application, including gene editing or stem cell transplantations. Strong overall correlation was observed between the liquid and semi-solid CFU assays; though the latter generally identified more colonies, it was also associated with higher variability. Collectively, the liquid CFU assay reduced operator hands-on time as well as variability, thus providing a standardized approach compatible with an in vitro functional evaluation of human HSPCs, both in clinical and in a research context.

## Materials and Methods

### Collection of Human Blood Cells

Mobilized peripheral blood was collected by standard leukapheresis procedure at the Department of Clinical Immunology at Aarhus University Hospital, Aarhus, Denmark. Stem cells were mobilized to peripheral blood through self-administration of 960 µg g-CSF (Accofil, Accord Healthcare, Durham, NC, USA) subcutaneously once daily at 9 pm for four days leading up to harvest day. Donor Medical Evaluation (Work-up) and leukapheresis harvest procedure were performed following the JACIE and WMDA standards. Table S1 provides donor and harvest details. The final leukapheresis product was supplemented with autologous plasma and stored without agitation at 4 °C for a maximum of 24 h prior to enrichment of CD34 + HSPC.

### Enrichment of CD34 + HSPCs

CD34 + HSPCs were isolated from the leukapheresis product by immunomagnetic separation, either in small or large scale. In small scale, the isolation of CD34 + HSPCs was performed using an anti-CD34 MicroBeads Kit (CD34 MicroBeads Kit, Human, cat: 130-046-702, Miltenyi Biotec, Bergisch Gladbach, Germany), according to the manufacturer’s instructions, in combination with MS columns (cat: 130-042-201, Milenyi Biotec) and the OctoMACS separator (cat: 130-042-109, Milenyi Biotec). In large scale, the isolation of CD34 + HSPCs was performed on the CliniMACS Prodigy^®^ (cat: 200-075-301, Miltenyi Biotec), using the “CD34 enrichment” standard procedure, according to the instructions by the manufacturer. For both scales of CD34 + isolation, the purity, yield, viability, and phenotype of the enriched cells were evaluated by flow cytometry. The CD34 + HSPCs were resuspended in CryoStor CS10 (cat: 07952, STEMCELL Technologies, Vancouver, Canada), followed by rate-controlled freezing, using a CoolCell™ LX Cell Freezing Container (Corning, Corning, NY, USA) placed at −80 °C for 24–48 h. The cells were subsequently stored in liquid nitrogen until further use.

### Liquid CFU Assay with a Flow Cytometry-Based Read-Out

For each CD34 + enriched sample, the differentiation and lineage potential of HSPCs were evaluated by standardized flow cytometry-based read-out, using the StemMACS HSC-CFU Assay Kit, Human (kindly provided by Miltenyi Biotec, cat: 130-125-042), according to the instructions of the manufacturer. In brief, CD34 + HSPCs were diluted to a concentration of 250 HSPCs/mL in StemSpan™ SFEM II Medium. 10 µL of cell suspension and 50 µL of StemMACS HSC-CFU Assay Medium were added to each well, in three round-bottom 96-well plates (cat: 163320, Nunclon Delta surface, Thermo Scientific). Cultures were maintained in a CO₂ incubator for 14 days at 37 °C and 5% CO_2_, with plates placed inside a small humidity chamber containing sterile water to minimize evaporation from the wells. The humidity chamber was checked regularly to ensure a sufficient water level. On day 14, the generated colonies in each well were stained with StemMACS HSC-CFU Assay Cocktail, containing anti-CD14-VioBlue, anti-CD15-APC, and anti-CD235a-PE. Flow cytometric acquisition was performed, using a NovoCyte Quanteon (Agilent, Santa Clara, CA, USA), using the following parameters: Plate type: 96-well (U bottom), Uptake volume: 50 µL, Sample volume: 100 µL, Flow rate: High, Mixing: 1 cycle/well (gentle), Rinse: 1 cycle/well, Max. events/sec: 1000. Gating of flow cytometric data was performed either using FlowJo™ Software (v10.9.0, BD Life Sciences, Franklin Lakes, NJ, USA) or NovoExpress Software (version 1.5.0., Agilent). In FlowJo, a template provided by the manufacturer of the StemMACS HSC-CFU Assay was used. Subsequent colony assignment, based on expression pattern of the gated populations, was performed in Excel 2016 (Microsoft Corporation, Redmond, WA, USA), resulting in distribution into colony types defined as BFU-E, CFU-GEMM, CFU-GM, CFU-M, CFU-G (Table 1). The minimum number of events required to be included in the data analysis, was 250 events/well and 35 positive events for each detected marker, CD14, CD15, and CD235a.

**Table 1:** Assay limits and recommended limits, yielding detection parameters for colony assignment in liquid CFU assay, based on cellular expression of CD14, CD15, and CD235a

Phenotype	Colony type	Assay limits			Recommended limits		
		% CD15^+^	% CD14^+^	% CD235a^+^	% CD15^+^	% CD14^+^	% CD235a^+^
CD15 + CD14 + CD235A+	**CFU-GEMM**	≥ 15	≥ 15	≥ 20	> 15	> 15	> 20
CD15 + CD14 + CD235a-	**CFU-GM**	≥ 30	≥ 30		> 10	> 20	
CD15- CD14 + CD235a-	**CFU-M**		≥ 50			> 19	
CD15 + CD14- CD235a-	**CFU-G**	≥ 50			> 19		
CD15- CD14- CD235a+	**BFU-E**			≥ 50			> 20
CD15 + CD14- CD235a+	**CFU-G & BFU-E**	≥ 50		≥ 50	> 15		> 20

Detected flow cytometric events were divided into five distinct colony types, based on their phenotypic characterisation. Assay limit parameters for stringent analyses are defined in the manufacturer´s instructions (StemMACS HSC-CFU Assay Kit, Human, Miltenyi Biotec). Recommended limits, based on assay optimization, are also provided by the assay manufacturer

### MethoCult Semi-Solid CFU Assay

A total of 100 or 250 CD34 + HSPCs were seeded in 35-mm discs in 1 ml of MethoCult H4435 (cat: 04435, STEMCELL Technologies), supplemented with penicillin-streptomycin and allowed to incubate for 14 days at 37 °C, in 95% humidity and 5% CO_2_. For each cell density, seeding was performed in triplicate discs. Colony-forming cells were counted and scored by morphology, using a brightfield microscope (CKS41SF, Olympus Corporation, Tokyo, Japan) as BFU-E, CFU-GEMM, CFU-GM, CFU-M, CFU-G, following criteria for colony identification [9].

### Colony Picking for Cross-Assay Correlation

For each colony type, (BFU-E, CFU-G, CFU-M, CFU-GM, CFU-GEMM), a total of 5 colonies were selected from the semi-solid MethoCult CFU assay and supporting colony images were acquired using a brightfield microscope. Each colony was transferred to a well in a 96-well plate with round bottom, containing StemSpan™ SFEM II Medium, and placed in a CO_2_-incubator at 37 °C and 5% CO_2_. After 12 h, the colonies were stained with the StemMACS HSC-CFU Assay Cocktail (cat: 130-125-042, Miltenyi Biotec), gently resuspended, and analysed by flow cytometry (NovoCyte Quanteon, Agilent), following the instructions of the assay kit manufacturer.

### Cellular Phenotyping of HSPCs Using Flow Cytometry

For absolute HSPC counts, anti-CD34-PE (clone 581, cat: 555822) and anti-CD45-FITC (clone 2D1, cat: 345808)(BD Biosciences, Franklin Lakes, NJ, USA) were used. The 7-Aminoactinomycin D (7AAD) DNA stain (cat: 7240-37-1, AAT Bioquest, Sunnyvale, CA, USA) was used to identify non-viable cells. Cells were resuspended in PBS/BSA buffer (0.1% BSA) prior to staining. For the absolute count of HSPCs, cells were stained with 7AAD, anti-CD34, and anti-CD45 antibodies for 15 min at RT. The acquisition of stained cells was either performed on a NovoCyte 3000 (Agilent) or a NovoCyte Quanteon (Agilent), using the NovoExpress Software (version 1.5.0). Calibration of the cytometers was performed daily, using the NovoCyte QC particles (cat: 8000004, Agilent). Compensation was performed monthly, using CompBeads, (Anti-Mouse Ig, K, cat: 51–90-9001229 and Negative Control, cat: 51–90-9001291, BD Biosciences). All gating strategies for absolute counting and cellular phenotyping were based on Fluorescence Minus One (FMO) controls, with a maximum of 0.2% positive events in the double-positive quadrant.

## Statistics

Statistical analyses were conducted using GraphPad Prism for macOS (version 10.2.3, GraphPad Software, Boston, Massachusetts, USA), unless otherwise specified. Due to limited sample sizes, statistical assumptions of normality and homogeneity of variance could not be reliably assessed; therefore, non-parametric methods were used throughout. To evaluate reproducibility of the liquid CFU assay, Friedman’s test was used to assess colony distribution, while the Kruskal-Wallis test was applied to evaluate colony potential; both were followed by Dunn’s multiple comparisons test for post hoc pairwise analysis. For analyses involving biological variation, statistical comparison of colony distributions was performed in R (version 4.4.2, R Foundation for Statistical Computing, Vienna, Austria). Aligned Rank Transformation (ART) was applied using the ARTool package to facilitate non-parametric analysis while meeting the assumptions of normality and homogeneity of variance. An analysis of variance (ANOVA) was then conducted on the transformed data to evaluate significant effects in the factorial model. Colony potential was again evaluated using the Kruskal-Wallis test, followed by Dunn’s multiple comparisons test. The agreement between gating strategies in the liquid CFU assay was evaluated using contingency analysis in JMP Software (Pro version 18.02, SAS Institute Inc., Cary, NC, USA). To assess correlation between the liquid CFU assay and the traditional semi-solid CFU assay, statistical significance was determined using multiple Mann-Whitney tests. Results are presented as mean + standard error of the mean (SEM), unless otherwise indicated. *p*-values ≤ 0.05 were considered statistically significant. Statistical significance is denoted as follows: *p* ≤ 0.05 (*), *p* ≤ 0.001 (**), *p* ≤ 0.0001 (***).

## Study Approval

G-SCF-mobilized cells were collected by standard leukapheresis procedure from enrolled study participants at the Department of Clinical Immunology at Aarhus University Hospital, Aarhus, Denmark under a protocol approved by The Central Denmark Region Committees on Health Research Ethics with approval number 1–10-72–144-19. Prior to the study inclusion, written informed consent was obtained from each participant. Excess material collected from patients undergoing autologous HSC transplantation was applied under similar conditions to evaluate the assay in patient material. Following inclusion, study data were collected and managed using REDCap electronic data capture tools hosted at Aarhus University, Aarhus, Denmark [10, 11]. All study participants were pseudo-anonymized and were subsequently only identified by a unique study number.

## Results

### Flow Cytometric Strategy for Identification of Colony Formation in Liquid CFU Assay

**Fig. 1:**
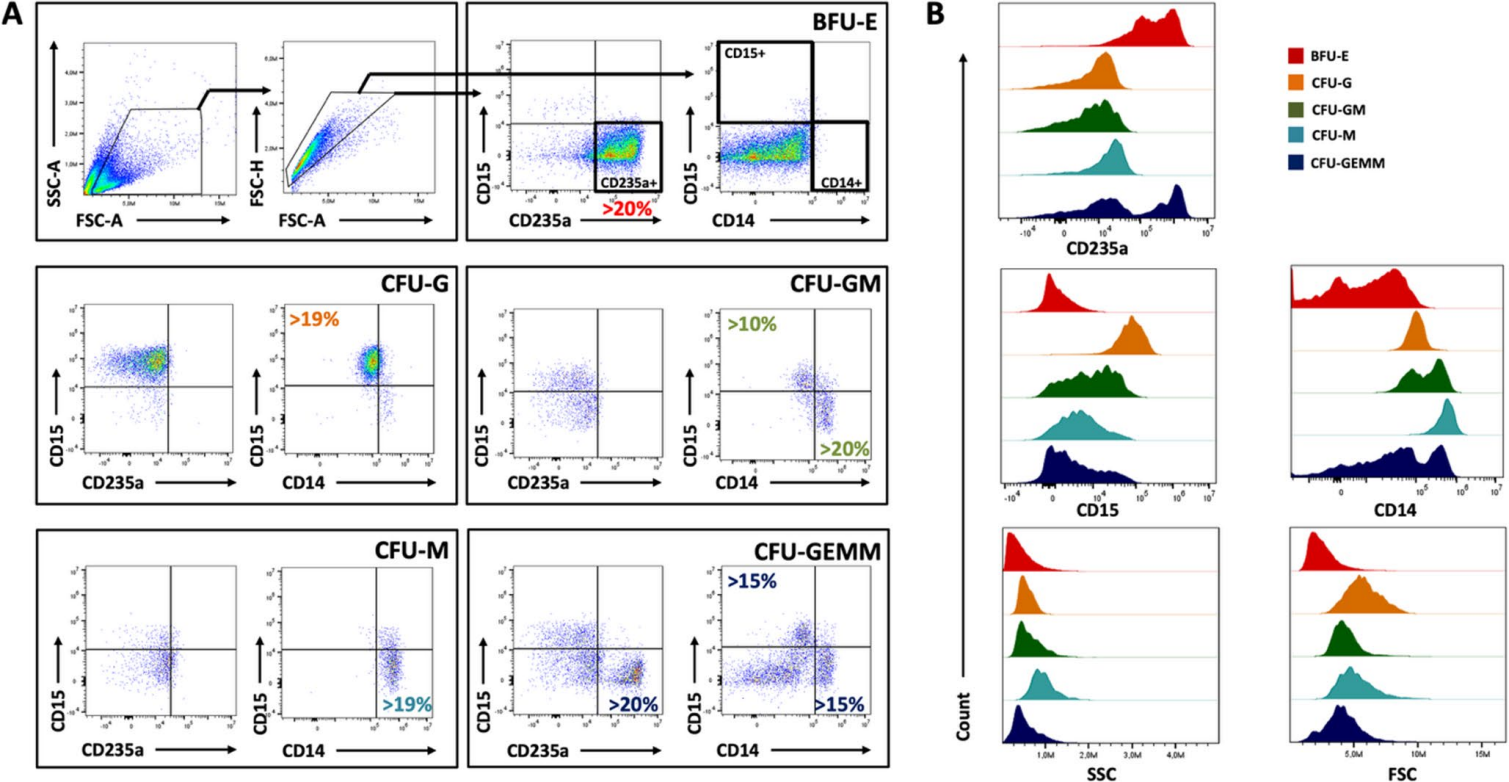
Gating strategy in FlowJo. (**A**) Representative plots illustrating the gating strategy to define five different colony types in a liquid CFU assay, using a gating template specified in FlowJo. After excluding debris and doublets, quadrant gates in two bivariate plots, displaying various combinations of CD14, CD15, and CD235a, were applied to classify CFUs. Gate adjustments for CD14 and CD15 were performed in a well containing predominantly CD235 + events (BFU-E). Recommended limits for colony assignment (Table 1) are shown for each colony type. (**B**) Representative histograms showing the distribution of the three evaluated surface markers as well as scatter parameters for each defined colony type

To assess the proliferation and lineage potential of enriched CD34 + HSPCs after 14 days in a methylcellulose-free liquid CFU assay, cells were stained directly in the wells followed by flow cytometric acquisition. The expression of three different surface markers, CD14, CD15, and CD235a, were used to define lineage specific differentiation. The data analysis entailed an initial gating on a representative well containing exclusively CD235a + erythroid events, allowing for reliable adjustment of CD14 + and CD15 + gates by exploiting the known absence of myeloid markers on erythroid cells. These calibrated gates were then uniformly applied across all 288 wells to enable high-throughput analysis. Event counts and percentwise expression of all markers were used to classify distinct colony types, defined by a set of detection parameters (Table 1). To evaluate how analysis software and gating strategy may influence data interpretation, two distinct gating workflows were evaluated. This dual approach served both to validate the robustness of the assay across platforms and to assess the extent to which gating strategy impacts downstream results. The first strategy, developed in FlowJo (Fig. 1, Panel A), followed a gating template provided by the manufacturer of the liquid CFU assay kit. This reflects the standard method likely to be adopted by users applying the assay as recommended.

**Fig. 2:**
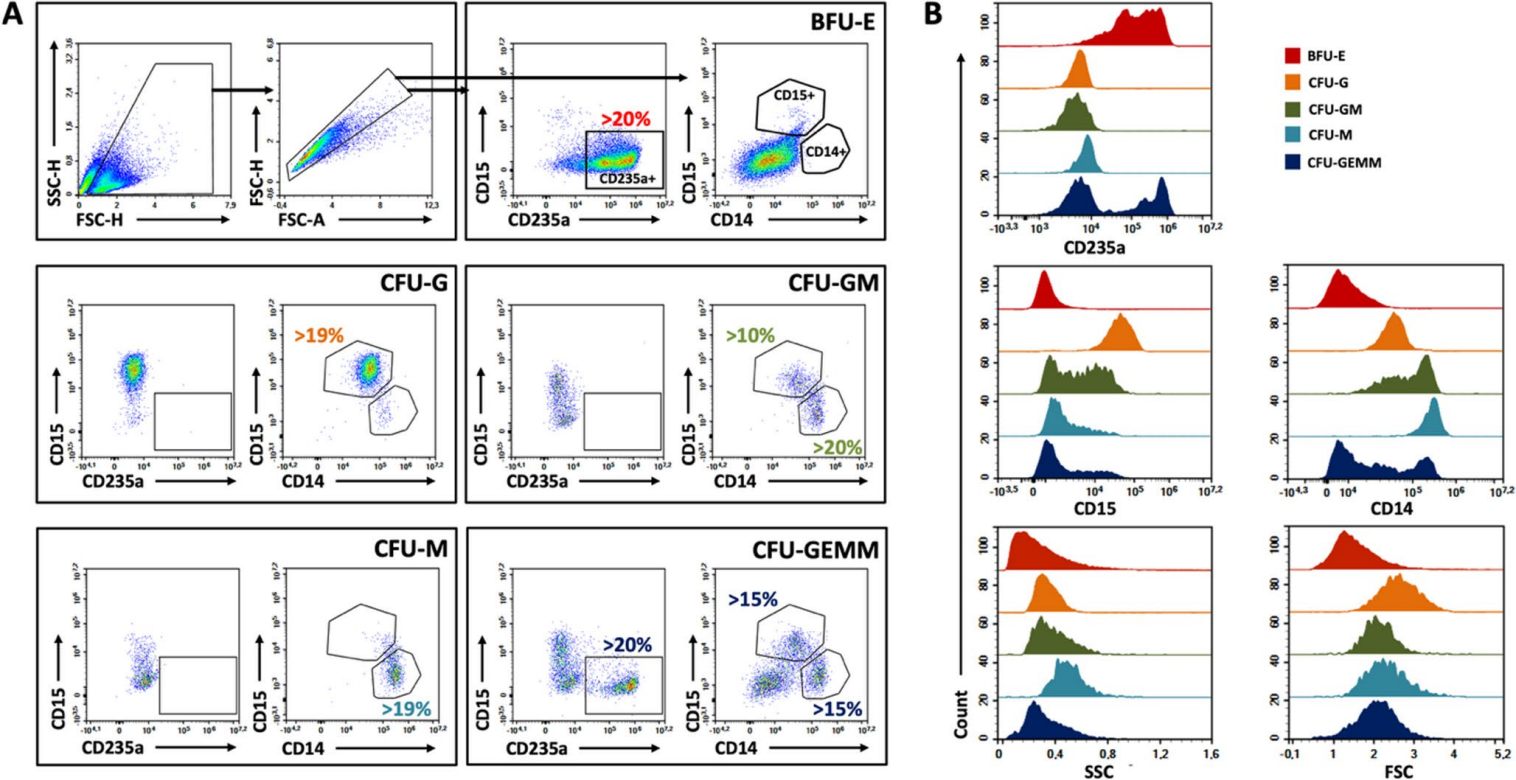
Gating strategy in NovoExpress. (**A**) Representative plots illustrating the gating strategy to define five different colony types in a liquid CFU assay, using a gating template detailed in NovoExpress on the same samples as illustrated in Fig. 1. Debris and doublets were initially excluded, after which rectangular or polygon gates in two different bivariate plots, displaying various combinations of CD14, CD15, and CD235a, were applied to classify CFUs. Gate adjustments for CD14 and CD15 positivity were performed in a well containing predominantly CD235 + events (BFU-E). Recommended limits for colony assignment (Table 1) are shown for each colony type. (**B**) Representative histograms showing the distribution of the three evaluated surface markers and two scatter parameters for each defined colony type

The second strategy was established in NovoExpress (Fig. 2, Panel A), the instrument-specific acquisition and analysis software for the NovoCyte flow cytometer used to generate all data in the current study. This gating approach was independently optimized to reflect the specific characteristics of the acquired data. For both gating workflows, the defined colony type displayed distinct surface marker combinations and scatter properties (Figs. 1 and 2, Panel B), while a negative unstained control demonstrated minimal background fluorescence and supported the specificity of antibody-derived signals (Figure S1).

### Performance of Liquid CFU Assay

**Fig. 3:**
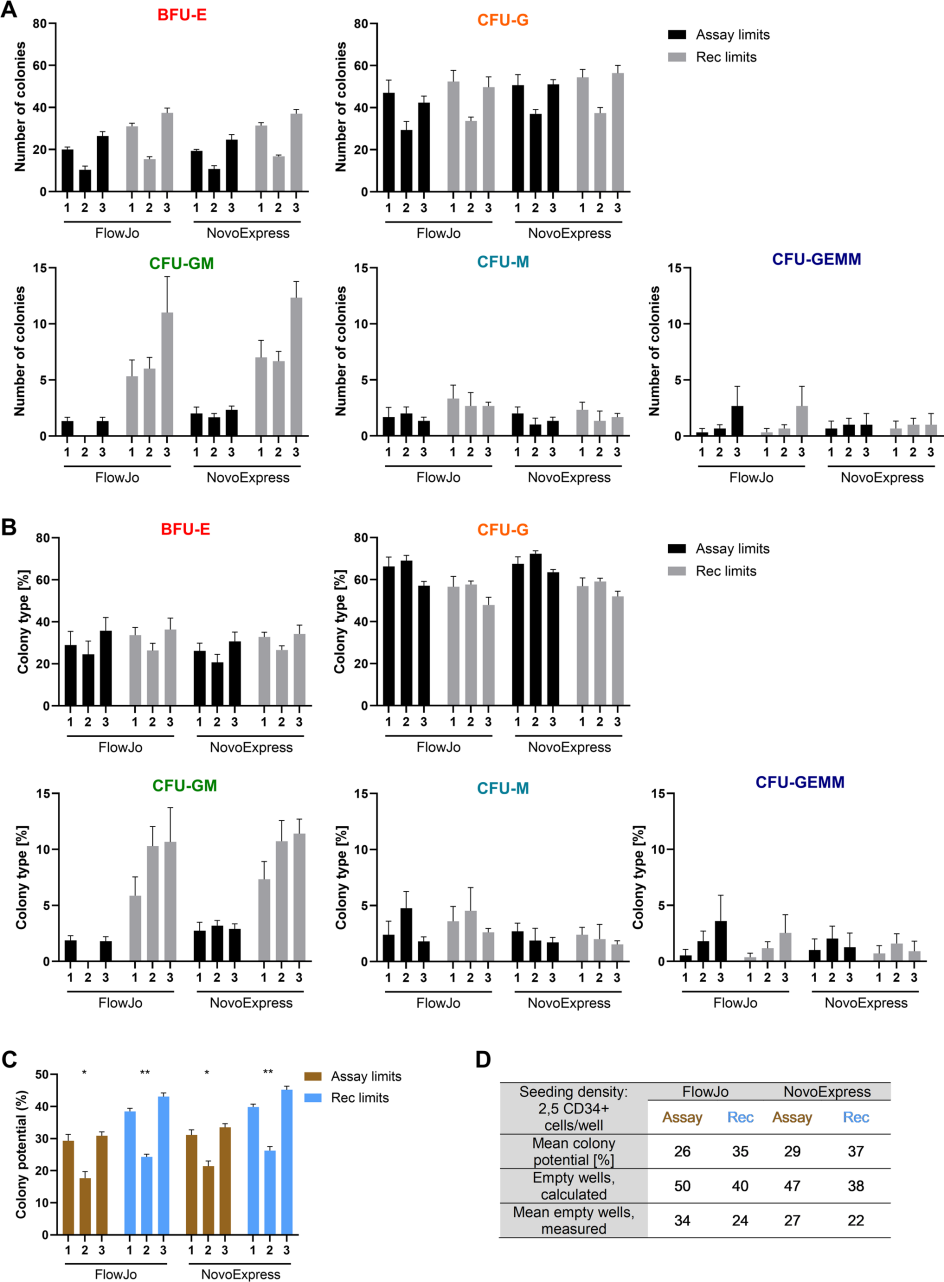
Liquid CFU assay yields reproducible results. Three different operators repeated the liquid CFU assay at different time points, using cells from the same stem cell donor (Donor 4). Analyses of acquired flow cytometric data included two different gating strategies (FlowJo, NovoExpress) and two separate colony assignment parameters (Assay limits, Recommended (Rec) limits). Counts (**A**) and percentwise distribution (**B**) of identified colony types were determined. **C**) Colony potential, calculated as the number of colonies/number of seeded cells. **D**) The expected number of empty wells was determined by Poisson distribution, using the mean colony potential and the seeding density. Statistical significance was determined by either Friedmans test (Panel A and B) or Kruskal-Wallis test (Panel C); (*); *p* ≤ 0.05, (**); *p* ≤ 0.001, (***); *p* ≤ 0.0001

Using enriched CD34 + HSPCs from one healthy stem cell donor, the assay performance and precision were evaluated by three independent experiments. Each experiment was performed by a different operator and at separate time points to maximize possible variation. At culture start, the cell viability was 97.1% ± 1.4 and the CD34 + frequency of the seeded cells were 98.8% ± 0.6, ensuring comparable starting points across experiments. For each gating strategy (Figs. 1 and 2), assay limits as well as recommended limits (Table 1) were applied as detection parameters to assign colonies, consequently resulting in a total of four different read-outs for colony counts and CFU distributions. Initially, the intra-assay variation was evaluated by comparing the total number of colonies detected across the three plates in each experiment. Based on intra-assay %CV values of the obtained total colony counts (Table S2), mean values of triplicate measurements were used for the remaining result evaluations. The lowest variation was observed for the recommended colony assignment parameters (Table S2). Next, the assay reproducibility, or inter-assay variation, was assessed by comparing results obtained from each of the three independent experiments. By comparing the absolute number of identified colonies of a specific type (Fig. 3A), comparable results were detected between operators, regardless of initial gating strategy and colony assignment parameters. Similarly, no significant differences were found for the percentwise distribution of colony types, which additionally provided a more stable read-out than the absolute colony type numbers (Fig. 3B).

Another metric to evaluate the performance of the assay is the colony potential, defined as the total number of colonies observed in relation to the number of seeded cells at culture start. Overall, the colony potential did display a degree of inter-assay variation (Fig. 3C), with the colony assignments based on the assay limits displaying overall significant differences (FlowJo: *p* = 0.050; NovoExpress: *p* = 0.032), yet no significant differences emerged in the pairwise comparisons. When using the recommended detection parameters, an overall statistically significant difference was observed with the Kruskal-Wallis test (FlowJo: *p* = 0.004; NovoExpress: *p* = 0.004), while the post hoc pairwise comparisons revealed a significant difference between operator 2 and 3 (FlowJo: *p* = 0.021; NovoExpress: *p* = 0.020). The largest colony potential was identified with the recommended limits, reflecting 30–35% more colonies generally identified with these parameters, compared to the results obtained with the assay limits. However, the observed mean colony potentials (Fig. 3D) ranged close to the maximum potential of 30%, as suggested by the assay manufacturer. The applied seeding density and an appropriate colony potential should ensure a proper balance between empty wells and wells with single CFU, aiming to avoid a surplus of wells with two different CFUs. Based on Poisson distribution, the number of calculated empty wells was slightly higher that the measured numbers, indicating a possible advantage of slightly lowering the seeding density. Nevertheless, both the number of each colony type and their percentwise distribution were reproducible, pointing to a somewhat limited effect of this in the presented study.

### Analytical Detection Width of Liquid CFU Assay

**Fig. 4:**
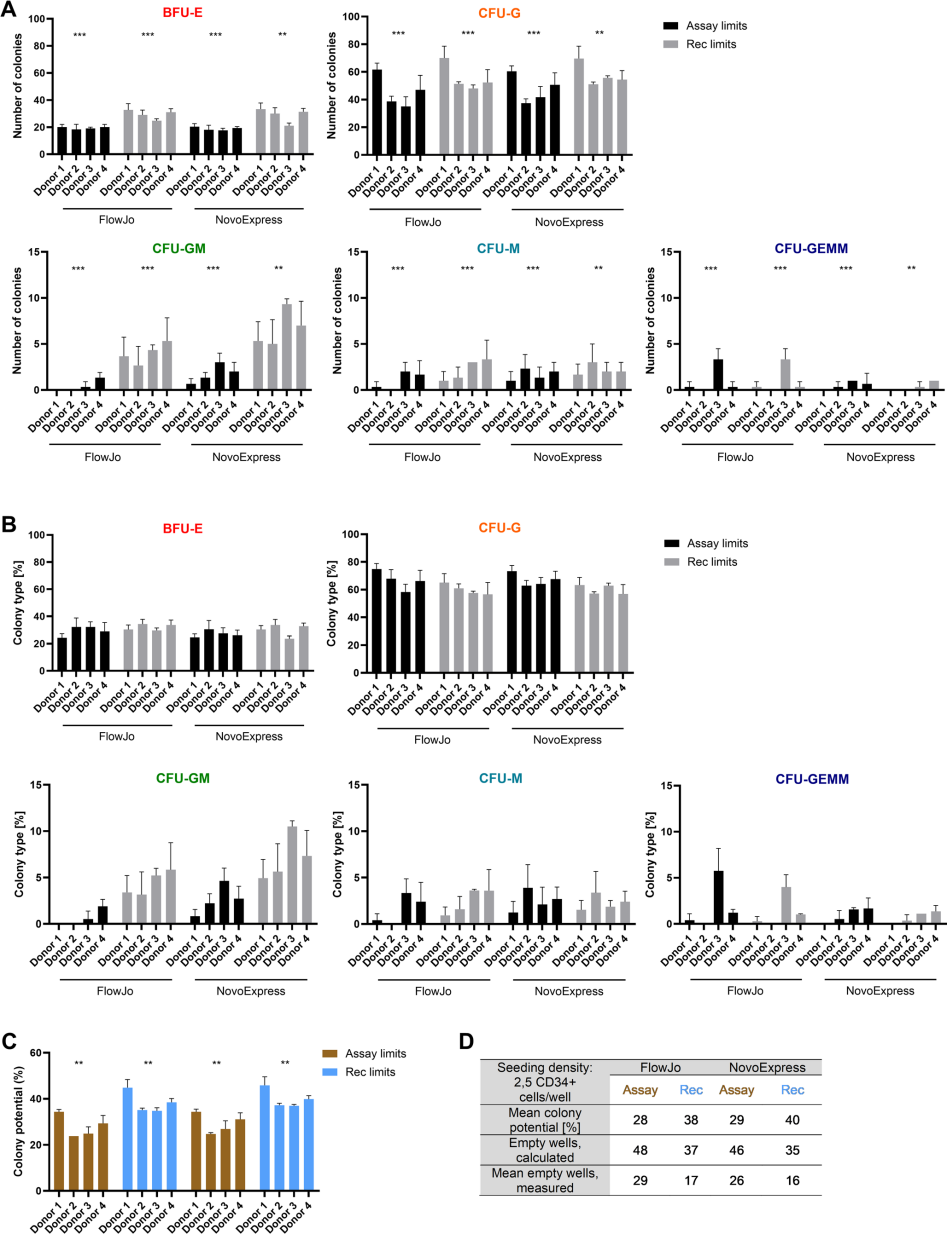
CD34 + cells from healthy donors produce comparable colony type profiles with liquid CFU assay. Using CD34 + cells from four different healthy stem cells donors, liquid CFU assays were prepared by the same operator. Analyses of acquired flow cytometric data included two different gating strategies (FlowJo, NovoExpress) and two separate colony assignment parameters (Assay limits, Recommended (Rec) limits). Counts (**A**) and percentwise distribution (**B**) of identified colony types were determined. **C**) Colony potential, calculated as the number of colonies/number of seeded cells. **D**) The expected number of empty wells was determined by Poisson distribution, using the mean colony potential and the seeding density. Statistical significance was determined by either non-parametric Aligned Rank Transform for factorial ANOVA (Panel A and B) or Kruskal-Wallis test (Panel C); (*); *p* ≤ 0.05, (**); *p* ≤ 0.001, (***); *p* ≤ 0.0001

After assessing the assay performance, we next evaluated the effects of biological variation, using CD34 + HSPCs from four different healthy stem cell donors. At culture start, the cell viability was 97.1% ± 1.0 and the CD34 + frequency of the seeded cells were 96.0% ± 4.7. Following the same data analysis strategy as described for the reproducibility data, the two different gating strategies illustrated in Figs. 1 and 2 were applied on the acquired flow cytometry data, followed by colony assignment of the gated populations, using assay limits and recommended limits (Table 1) as detection parameters. First, we evaluated the effect of donor variation on the absolute number of each type of colony arising with the four analysis combinations. For all combinations, a statistically significant effect of donor variation was observed (Fig. 4A). Across all donors, a majority of CFU-G and BFU-E colonies were identified, while only very few CFU-GEMMs were observed in all samples. Additionally, more colonies were generally identified with the recommended colony assignment parameters compared to the more stringent analysis, using the assay limits. In contrast to this, a more conserved percentwise distribution of colony types was identified in all four analysis combinations (Fig. 4B), creating comparable colony distribution profiles for the included donors. Furthermore, the effect of biological variation on the percentage of colony type was not statistically significant, pointing to this as a more stable assay read-out over the absolute number of colonies, which is similar to the observations from the reproducibility evaluation (Fig. 3).

Focusing on the colony potential, the donor effect was significant across all analysis combinations (Fig. 4C). For the assay limits (FlowJo: *p* = 0.006; NovoExpress: *p* = 0.010), the pairwise comparisons showed a significant difference between Donor 1 and 2, when combined with the FlowJo gating strategy (*p* = 0.037). For the recommended limits (FlowJo: *p* = 0.002; NovoExpress: *p* = 0.001), the pairwise comparisons showed a significant difference between Donor 1 and 3, regardless of initial gating strategy (FlowJo: *p* = 0.046; NovoExpress: *p* = 0.038). Furthermore, the largest colony potential was identified with the recommended limits, following a previously detected pattern (Fig. 3C). Finally, the mean colony potentials for all analysis combinations were also similar to those identified for the assay performance test, ranging near the recommended maximum of 30%. While the number of observed empty wells in all cases was smaller than calculated expectation (Fig. 4D), a similar percentwise distribution of colonies was identified across donors, substantiating the robustness of this read-out over the colony counts.

To further elaborate on the effects of gating strategy and colony assignment parameters, a contingency analysis was performed, evaluating the degree of conformity. Starting with the total number of colonies, the more stringent analysis, using the assay limits, identified fewer colonies than the recommended colony assignment (Figure S2; Tables S3 and S4), correlating with the lowered threshold for identification used in the latter approach (Table 1). The lowest total colony count was found with the combination of FlowJo gating and assay limits for colony assignment. An additional effect of lowering the threshold for identification was more double assignments (BFU-E and CFU-G), while simultaneously increasing the number of identified CFU-GM and CFU-M, which can also be observed in Fig. 4A. A similar effect could not be detected for the overall identification of CFU-GEMMs. In addition to the contingency analysis, Kappa coefficients were calculated for each comparison (Tables S3 and S4), to enumerate the strength of agreement. This yielded a Kappa coefficient of 0.65 and 0.61 for the comparison of the FlowJo and NovoExpress gated data, respectively. This can be interpreted into substantial agreement, using the scale of Landis & Koch, which is defined as a Kappa between 0.61 and 0.8, while a Kappa of 0.41–0.6 translates into moderate agreement [12].

### Correlation Between Liquid CFU Assay and Traditional Semi-Solid CFU Assay

**Fig. 5:**
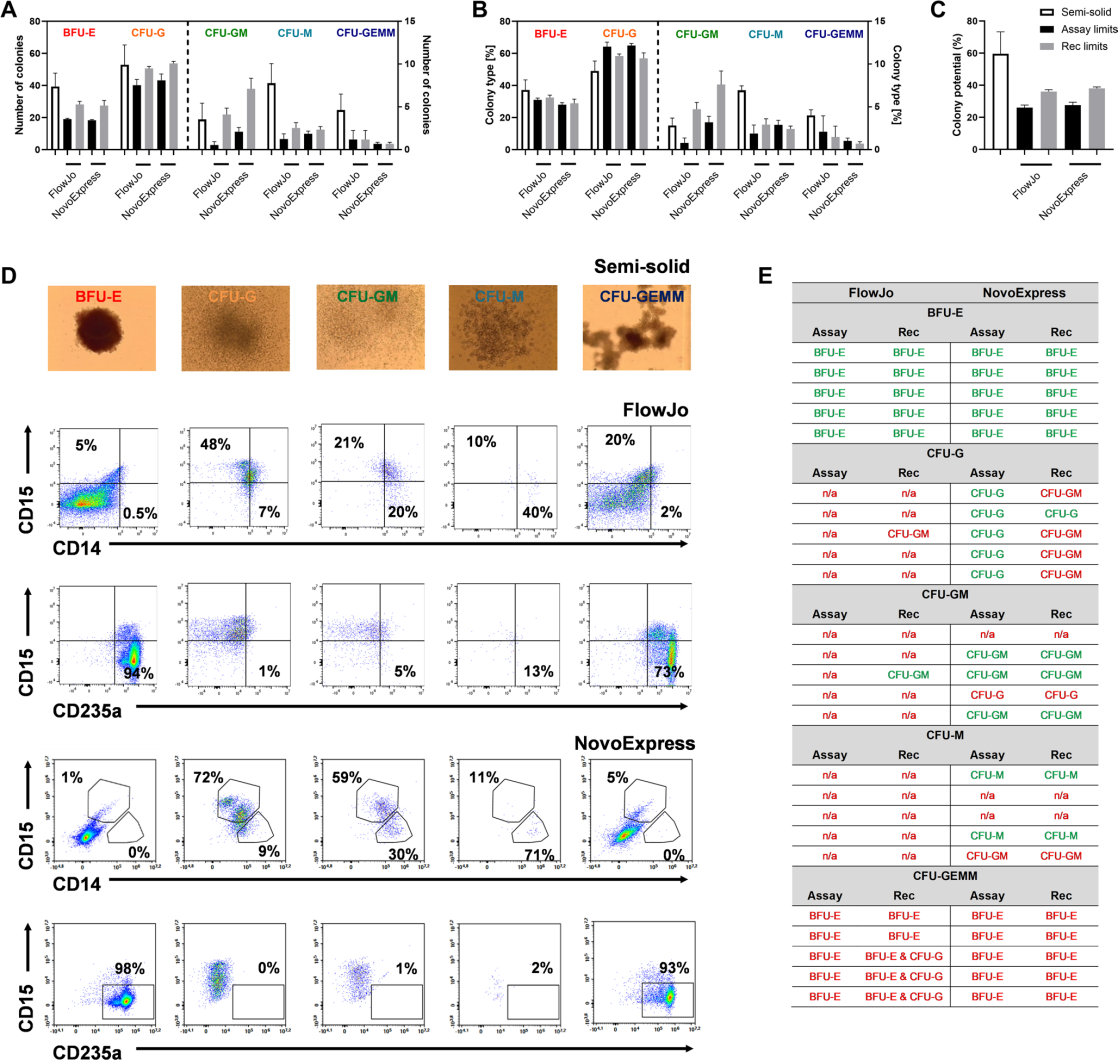
Semi-solid CFU assay correlates with liquid CFU assay. CD34 + cells from three out of four healthy stem cells donors evaluated in the liquid CFU assay (Fig. 4) were seeded in a semi-solid CFU assay. The resulting colonies were scored to different colony types after morphological evaluation. The results were compared to those obtained for the three donors in the liquid CFU assay (Donor 2, 3, and 4; Fig. 4). Counts (**A**) and percentwise distribution (**B**) of identified colony types were determined. For the liquid CFU assay, data analyses of acquired flow cytometric data included two different gating strategies (FlowJo, NovoExpress) and two separate colony assignment parameters (Assay limits, Recommended (Rec) limits). **C**) Colony potential, calculated as the number of colonies/number of seeded cells. **D**) For cross-assay correlation, 5 colonies of each colony type were picked from the semi-solid assay of Donor 4 and analyzed by the same flow cytometric setup used in the liquid CFU assay. Representative microscopic images of each colony type picked and the corresponding plots from the subsequent flow cytometric analysis. Gating of data was performed in FlowJo and NovoExpress softwares. **E**) Agreement (green) or disagreement (red) between colony assignments of the 5 picked colonies of each type from the semi-solid CFU assay and subsequent phenotypical analyses by flow cytometry. Statistical significance was determined by Multiple Mann-Whitney tests (Panels A - C); (*); *p* ≤ 0.05, (**); *p* ≤ 0.001, (***); *p* ≤ 0.0001

The semi-solid CFU assay is widely used for in vitro assessment of the multi-lineage capacity of HSPCs and consequently served as a suitable benchmark assay for comparing the liquid CFU assay tested in the present study. To allow for direct comparison of the degree of correlation between the outputs of the two functional assays, enriched CD34 + HSPCs from three of the four healthy donors used in the liquid CFU assay (Donors 2–4) were also evaluated in the semi-solid CFU assay. At culture start, the cell viability was 92.6% ± 0.3, while the CD34 + frequency of the seeded cells was 97.4% ± 1.8. Evaluating the intra-assay variation of the total number of colonies detected within technical triplicates in each assay demonstrated considerably lower %CV values for the liquid CFU assay compared to the semi-solid counterpart (Table S5). The lowest intra-assay variation was achieved in the liquid CFU assay, when using the recommended colony assignment parameters, which was similarly noted in the reproducibility assessment (Table S2). However, strong overall correlation between the two CFU assays was detected, both for the absolute count of each individual colony type and for the resulting percentwise distribution (Fig. 5A and B). Generally, the semi-solid assay identified more colonies than the liquid assay, resulting in higher colony potential in the traditional CFU assay (Fig. 5C). Nevertheless, no statistically significant differences were observed for the measured colony potentials, when comparing all possible combinations.

To further extend the correlation assessment, a direct cross-assay evaluation was performed. Five colonies of each type (BFU-E, CFU-G, CFU-GM, CFU-M, and CFU-GEMM) were identified based on their morphology, after which each colony was picked from the semi-solid assay and subsequently stained with the antibody mix from the liquid CFU assay, to evaluate their surface expression of CD14, CD15, and CD235a (Fig. 5D). For BFU-E colonies, cross-assay correlation was complete, regardless of gating strategy and colony assignment parameters (Fig. 5E). For CFU-G colonies, only the NovoExpress gated data combined with the assay limits assigned colonies identical to the morphological evaluation. Using the lowered threshold for particularly CD14 + in the recommended limits, converted several CFU-G colonies to CFU-GM. This is a feature also illustrated from the contingency analyses (Tables S3 and S4; Figure S2). More CFU-GM colonies were also identified cross-assay using the NovoExpress based gating strategy, possibly correlated to the fewer events included the relevant gates in the FlowJo strategy (Tables S6 and S7). For cross-assay detection of CFU-M colonies, NovoExpress gated data generally performed better than the FlowJo counterpart, despite few events in all cases. However, CFU-M colonies were generally not very frequently identified in the liquid CFU assays presented in this study (Figs. 3 and 4). The colonies identified as CFU-GEMMs in the semi-solid assay were all cross-assay identified as a BFU-E containing colony, when evaluating surface marker expression (Fig. 5E). As a main feature, CFU-GEMMs were largely identified in the semi-solid assay compared to the liquid CFU assay (Figs. 5A and B). Conversely, the expression pattern of CD14, CD15, and CD235a, as well as light scatter characteristics, of colonies identified as CFU-GEMM in the semi-solid assay were more similar to colonies, which the liquid CFU assay predominantly identified as BFU-E colonies (Figure S3). This may point to either differences in the scoring schemes of the two different CFU assays or a preferential support of different colony types within each assay.

Taken all evaluation steps together, the aim was to assess if a new liquid CFU assay with a flow cytometric read-out could be applied for in vitro assessment of the proliferation and lineage potential of enriched CD34 + HSPCs. These steps included evaluation of reproducibility, assay detection width, and correlation with the widely used semi-solid CFU assay. The liquid CFU assay proved reproducible and yielded comparable colony profiles across healthy stem cell donors. The lowest variation was observed when using the recommended colony assignment and the percentwise distribution of the colony types provided the most stable read-out. In the cross-assay correlation, the presented gating strategy in NovoExpress offered more precise gating than the FlowJo strategy optimized by the manufacturer of the liquid CFU assay. Despite a large degree of conformity between the colony assignment schemes, the choice of colony assignment parameters has a larger impact on the final results than the effect of the gating strategy. Although the NovoExpress gating combined with the assay limits for colony assignment demonstrated the highest degree of cross-assay correlation, combining the NovoExpress gating with the recommended limits resulted in the lowest assay variation, both in terms of technical and biological variation (Tables S2 and S5). Hence, when balancing out all evaluated features, the NovoExpress gating in combination with the recommended assay limits entails the chosen strategy to employ.

### Extending the Liquid CFU Assay to Other Relevant Samples

**Fig. 6:**
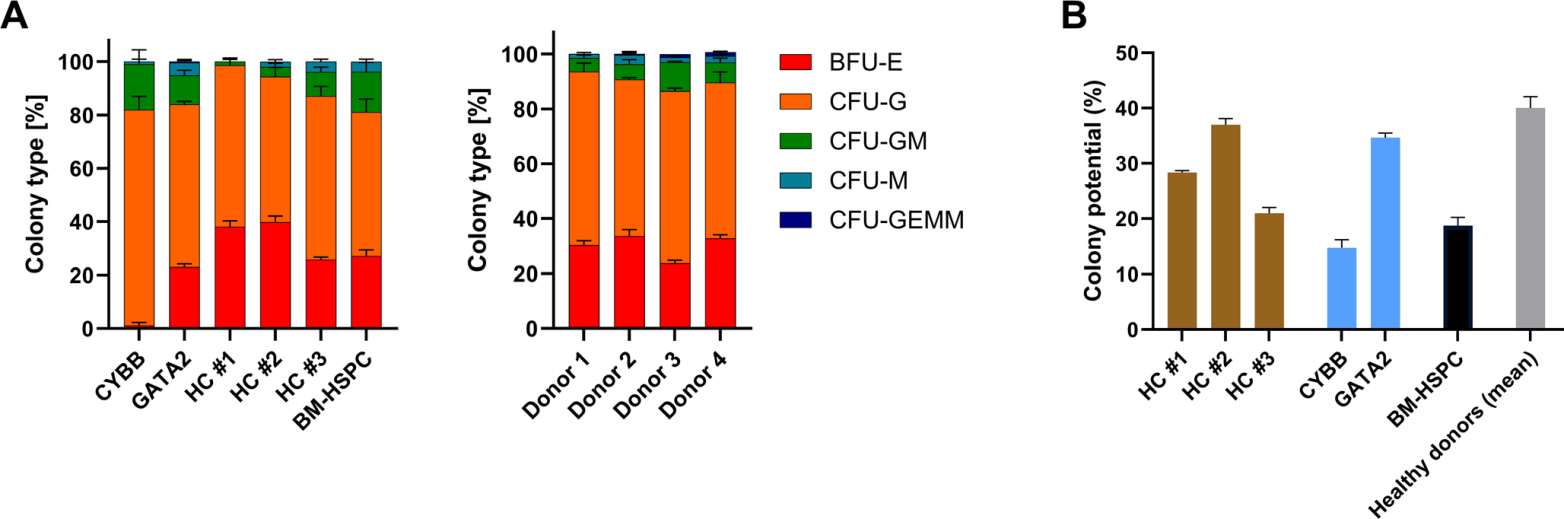
Liquid CFU assay compatible with different sample types. CD34 + cells from a selection of relevant sample types were evaluated in the liquid CFU assay, followed by data analysis using the selected parameters (NovoExpress gated; Recommended limits). All CD34 + cells were from mobilized peripheral blood, except the bone marrow (BM)-HSPCs. Patient samples were related to known immunodeficiency-causing mutations in *CYBB* and *GATA2* or to haematological cancers (HC; undisclosed type). (**A**) The identified colony types in all samples, including CD34 + cells from four healthy donors (also presented in Fig. 4). (**B**) Colony potential, calculated as the number of colonies/number of seeded cells

Assay performance and identification of critical points in the data analysis of the liquid CFU assay were evaluated using enriched CD34 + HSPCs from healthy stem cell donors. However, to extend the applicability of the assay, we investigated other relevant sample types, representing both patient samples and CD34 + HSPCs from a tissue source other than mobilized peripheral blood. The clinically relevant samples related to immunodeficiencies (mutation in either *CYBB* and *GATA2*) or haematological cancers (HC), were collected from patients prior to autologous HSCT. At culture start, the cells enriched in small scale (HC #1–3 and BM-HSPC, Fig. 6), displayed a viability and CD34 + frequency of 97.4% ± 1.3 and 92.0% ± 4.7, respectively. For the samples enriched in large scale (CYBB, GATA2, and healthy donors), viability and CD34 + were 93.2% ± 5.1 and 95.9% ± 2.4, respectively. These new samples exhibited more diverse colony type profiles, compared to healthy stem cell donors (Fig. 6A), although still with a predominance of CFU-G and BFU-E colonies. The most noticeable difference was observed for the CD34 + HSPCs with a mutation in the *CYBB* gene. Here, identification of BFU-E colonies was almost absent, while a larger fraction of CFU-GM was produced, compared to all included samples except for CD34 + HSPCs from a healthy BM-HSPC donor. In relation to the colony potential, these bone marrow-derived cells displayed a lower potential than their counterparts from mobilized peripheral blood (Fig. 6B).

Overall, the liquid CFU assay and the chosen data analysis strategy supported the detection of varying multi-lineage and proliferative capacities of CD34 + HSPCs from both mobilized peripheral blood and bone marrow. The differences were observed across samples related to various haematological disease categories.

## Discussion

CFU assays are essential for evaluating the functional potential of HSPCs based on their capacity to form distinct colonies in vitro. Conventional CFU assays, typically performed in methylcellulose-based semi-solid medium, rely on labor-intensive manual colony classification by microscopy. To enhance standardization and reproducibility, we assessed a novel, commercially available liquid CFU assay that combines antibody staining with a flow cytometry analysis. This approach is readily adaptable to existing workflows in hematology and immunology, broadening its utility across diverse research and clinical settings.

To serve as a suitable methodological alternative, precision and robustness of the liquid CFU assay must be demonstrated. In evaluating assay precision, we demonstrated repeatability of the liquid CFU assay, as the intra-assay variation was low, regardless of the applied data analysis strategy. Of note, the intra-assay variation was considerably lower for the liquid CFU assay compared to the methylcellulose-based semi-solid assay, likely due to reduced operator bias in the flow cytometry-based readout. Similarly, the subjective nature of morphological assessment in the semi-solid assay could be minimized by adopting semi-automated imaging and colony scoring methods [4] or AI-based morphology recognition [13]. In addition to reduced intra-assay variation, the liquid CFU assay demonstrated high reproducibility, with minimal inter-assay differences in both absolute colony counts and the proportional distribution of colony types. Moreover, the analytical detection width of the assay was sufficient to capture the effects of biological variation within the absolute colony counts, regardless of gating strategy and colony assignment parameters. While the colony quantities significantly differed among the included healthy donors, the percentwise colony distribution profiles were comparable. To extend on that, the relative distribution of colony types may represent the broader lineage differentiation potential of HSPCs, whereas the absolute colony counts could serve as an indicator of proliferative capacity, a parameter potentially associated with functional endpoints such as in vivo engraftment kinetics. A correlation with in vivo data or therapeutic data could help to substantiate this theory, such as in other studies, where CFU counts and types, identified in the classic CFU assay, have been linked to clinical outcome [14–17].

Given that the liquid CFU assay read-out depends on fluorescence signal detection, optimal analysis requires precise resolution of individual fluorescence channels. This entails minimizing spectral overlap to ensure the reliable identification of discrete cell populations that are clearly distinguishable from background fluorescence. The antibody cocktail in the assay is provided with three different antibodies conjugated to the fluorochromes VioBlue (Ex, max: 400 nm; Em, max: 452 nm), PE (Ex, max: 565 nm; Em, max: 578 nm), and APC (Ex, max: 651 nm; Em, max: 660 nm). A widely used flow cytometry configuration are laser lines of 405, 488, and 640 nm, which facilitates excitation of each of the three fluorochromes with separate lasers. Using such a setup creates maximal signal separation and fluorescence compensation redundancy, facilitating an ease-of-use. We applied a configuration that also included a 561 nm laser, yielding comparable results. Moreover, the applied gating strategy should preferably be applicable with broadly available analysis software, and thus we tested two different platforms for this purpose. We found that the software optimized for the selected flow cytometer platform proved to be slightly more favorable in clearly defining the identified populations and provided the lowest intra-assay variation. However, the gating template created by the supplier of the liquid CFU assay, compatible with a widely used third-party software, also provided a suitable alternative, demonstrating broad applicability of this generalized template. This template may even be further refined, to better accommodate the contours of each detected population, thereby optimizing data analysis. In this study, software choice was treated as an analytical preference rather than a prespecified methodological factor. However, our agreement analyses demonstrate that detection-parameter thresholds had a stronger influence on colony assignment than the software platform itself. When thresholds were held constant, the two platforms showed high concordance, with Kappa coefficients of 0.86 and 0.89 for the Recommended and Assay limits, respectively, translating into almost perfect agreement [12]. In contrast, when the software was held constant and thresholds were varied, agreement declined (NovoExpress κ = 0.61; FlowJo κ = 0.65; Tables S3–S4). These findings indicate that threshold selection drives most of the observed variability, yet software choice is not entirely neutral and still contributes a measurable effect. Future work would benefit from treating software as an explicit methodological factor, including version control, preserved templates, and potentially FMO-anchored thresholding, to improve reproducibility. Finally, while our findings address analytical variability, the study was limited to a single cytometer configuration. Further validation should incorporate inter-instrument comparisons to ensure reproducibility across platforms, which is an essential requirement for broader implementation, especially in highly regulated setting.

In addition to analytical reproducibility, practical considerations such as time and cost influence assay adoption. To address this, we compared the liquid and semi-solid CFU formats across key operational metrics (Table S8). The liquid assay offers more predictable hands-on time, streamlined batch analysis, and reduced operator dependency, making it attractive for high-throughput and standardized workflows. However, these benefits come with the need for flow cytometer access, which may limit implementation. The semi-solid assay avoids instrument reliance and but demands extensive analyst time for colony identification; a challenge that becomes more pronounced for inexperienced operators. Taken together, though each approach has merits, laboratories should weigh these trade-offs in the context of available expertise and infrastructure.

While workflow efficiency determines the practical feasibility of an CFU assay, its core purpose is to enable reliable assessment of HSPC proliferation and lineage potential. In this regard, the parameters used for colony identification in the liquid CFU assay are critical, particularly the selection of surface markers for colony classification. The liquid CFU assay suggests classification of five distinct colony types - BFU-E, CFU-G, CFU-GM, CFU-M, and CFU-GEMM - based on differential surface expression of CD235a, CD14, and CD15. CD235a (Glycophorin A) is a well-established marker of erythroid differentiation, CD14 marks monocytes and macrophages, while CD15 is primarily expressed on granulocytes. This simplified marker strategy builds on a study by Görgens et al. (2013), in which expression of CD235a, CD14, CD15, CD45, and CD66b was correlated with colony morphology of CD34 + HSPCs in a semi-solid CFU assay using umbilical cord blood. While this minimal panel allowed classification of major myeloid lineages with reasonable resolution, its sufficiency in a liquid setting is less certain. In the present study, particularly identification of CFU-Ms, CFU-GEMMs, and, to some extent, CFU-GMs was considerably lower in the liquid assay compared to the semi-solid counterpart (Fig. 5A). A common feature of CFU-M, CFU-GEMM, and CFU-GM colonies is the expected presence of varying degrees of CD14-expressing cells, reported by previous studies [3, 18]. In our data, however, CD14 signal intensities were generally low, and colonies identified as CFU-M or CFU-GEMM in the semi-solid assays showed few or no CD14⁺ events in the cross-assay correlation (Fig. 5B). The antibody manufacturer confirmed no QC issues with the anti-CD14 reagent, leaving no clear methodological explanation for the reduced signal. These findings highlight the need to consider potential assay refinements, including the evaluation of alternative or supplementary surface markers for more reliable identification of monocytic populations. As an alternative, CD115 (CSF-1R) was shown to stain a similar population as CD14 [3] and could describe an earlier committed monocytic/macrophage lineage [19] than CD14 [20]. Hence, CD115 may serve as an alternative or supplementary monocytic/macrophage marker, if expression is supported in the liquid CFU assay. Likewise, inclusion of CD66b as an additional granulocyte marker to CD15 [18], could increase resolution and increase capture of rare or mixed-lineage progenitors such as CFU-GEMM. Adding to this, CD235a is considered a late-stage erythroid marker, while CD71 or CD117 might embrace the erythroid compartment to a larger extent, allowing an optimized identification and separation of BFU-E and CFU-GEMMs colonies, of which the latter has been demonstrated to be CD235a- [21, 22]. Beyond surface marker expression, the readily available forward (FSC) and side scatter (SSC) parameters (Figs. 1 and 2), reflecting cell size and granularity, might provide complementary information for more robust classification. However, the differences in scatter profiles between colony types were often subtle, which may limit their discriminatory power. While adding markers or additional tracking tools expand the analytical value of the assay, each added parameter increases the complexity of the fluorescence matrix, requiring careful compensation and potentially affecting sensitivity. Thus, multiplexing should be balanced against the goal of maintaining a simple and robust assay design. This study did not include experiments with alternative marker combinations suggested above. Nonetheless, systematic evaluation of varied marker panels represents an important next step to enhance lineage resolution and improve classification of particularly rare progenitor subsets.

As alluded to above, a key discrepancy observed in our study was the notably lower frequency of CFU-GEMM colonies identified in the liquid CFU assay compared to the semi-solid assay. This difference may partly reflect the subjective nature of manual colony identification in the semi-solid format. However, the number of CFU-GEMMs observed in the semi-solid assay (Fig. 5A) were slightly lower but still aligns with findings from previous studies, such as Görgens et al. (2013), which also identified multiple CFU-GEMMs using semi-solid CFU assays. A key feature of the semi-solid methylcellulose assay is the physical structure of the culturing matrix. This offers spatial segregation and localized cytokine gradients, potentially favoring the survival and detection of selected colony subsets [23]. In contrast, the absence of stromal support and a more homogeneous liquid culture may alter differentiation kinetics and marker expression profiles, promoting another lineage commitment. Moreover, culture media composition can influence colony output and lineage development in HSPC assays [3, 21] and while the listed cytokines for both assays are the same, concentrations are only disclosed by one of the manufacturers. Görgens et al. further demonstrated that colonies initially identified as BFU-E, based on CD235a expression and erythroid morphology, were, upon replating, revealed to possess multilineage potential characteristic of CFU-GEMMs. These misclassifications were especially prevalent in colonies derived from CD34 + CD133 + HSPCs, which for the healthy donors included in this study constituted over 80% of the input CD34 + population (Table S9). This suggests that a portion of the BFU-E colonies detected in the liquid assay may in fact represent CFU-GEMMs that failed to fully manifest their multilineage potential under the assay conditions or were misclassified due to a restricted marker use. Supporting this, we observed that the surface marker expression and light scatter profiles of CFU-GEMM colonies in the semi-solid assay closely resembled those of the predominant BFU-E-like events in the liquid assay (Figure S3). These findings highlight a possible limitation of the liquid CFU assay in detecting early multipotent progenitors, indicating that improved lineage resolution may require a more comprehensive marker panel, adjusted culture conditions, or application-specific optimization of colony classification thresholds.

Overall, our evaluation of a new liquid CFU assay demonstrates a robust and reproducible alternative to the classical semi-solid assay, offering several practical advantages. The liquid format enables a more standardized and potentially automatable workflow, with increased throughput, scalability, and reduced operator bias. Additionally, it allows for multiplexing and integration with downstream analyses, expanding its utility in both research and clinical contexts. However, this added flexibility introduces complexity, and any expansion of the marker panel must be carefully balanced against the assay’s intended simplicity and application. Conversely, this simplicity may constrain the assay resolution for identifying complex multilineage progenitors, such as CFU-GEMMs, which are more reliably captured in semi-solid assays through morphological evaluation. These findings highlight the broader challenge of designing a simplified in vitro system capable of fully representing the functional heterogeneity of HSPCs. Nonetheless, such complexity may not be necessary in all contexts, particularly in clinical settings, where quantification of specific progenitor subsets, such as the most primitive (CFU-GEMM and CFU-GM), may be sufficient for potency assays or outcome prediction. Importantly, we show that the liquid CFU assay performs consistently across a range of sample types, reinforcing its versatility. With further validation to confirm its biological and clinical relevance, this approach could support standardized potency evaluation in cell therapy manufacturing according to relevant guidelines [24, 25], while also serving as a valuable tool in research. Given the widespread integration of flow cytometry in hematology and clinical immunology, this liquid CFU assay represents a significant step toward a more scalable, standardized, and informative assessment of HSPC functionality.

## Conclusion

We demonstrate that a new liquid CFU assay delivers a fast, reproducible, and automation-ready alternative to semi-solid cultures for functional HSPC assessment. Its flow cytometry–based readout minimizes bias, streamlines analysis, and supports high-throughput, multiplexed workflows, making it equally valuable in research, clinical hematology, and immunology. While semi-solid formats still best resolve complex multilineage progenitors, the liquid assay provides robust quantification of defined subsets for most translational and clinical endpoints. With optimized marker panels and outcome validation, it could set a new standard for harmonized HSPC evaluation from bench to bedside. With its precision and scalability, the liquid CFU assay has the potential to replace subjective microscopic scoring with objective, flow cytometry–based quantification, to unite HSPC evaluation across laboratories and clinics, and accelerating progress from discovery to patient care.

## Supplementary Information

Below is the link to the electronic supplementary material.

Supplementary Material 1 (DOCX 3.02 MB)

## Data Availability

All data generated or analysed during this study are included in this published article and its supplementary information files.

## Abbreviations

ANOVA: Analysis of variance
BFU-E: Burst-forming unit-erythroid
BM: Bone marrow
CFU: Colony-forming unit
CFU-G: Colony-forming unit-granulocyte
CFU-GEMM: Colony-forming unit-granulocyte-erythroid-macrophage-megakaryocyte
CFU-GM: Colony-forming unit-granulocyte-macrophage
CFU-M: Colony-forming unit-macrophage
HC: Hematological cancer
HSPC: Hematopoietic stem and progenitor cells
