# Supplementary material for: Exploring a Flow Cytometry-Based CFU Assay for Functional Assessment of Human HSPCs: a Robust Alternative to Morphological Colony Evaluation
Source: Stem Cell Rev Rep. 2025 Dec 26;22(2):1007–22. doi: 10.1007/s12015-025-11045-w (PMC12858490; doi:10.1007/s12015-025-11045-w)
Supplement: Supplementary file 1 — Supplementary Material 1 (DOCX 3.02 MB) [file 12015_2025_11045_MOESM1_ESM.docx]

Table S1. Donor information and corresponding harvest information for their leukapheresis product.

|  | **Donor 1** | **Donor 2** | **Donor 3** | **Donor 4** |
| --- | --- | --- | --- | --- |
| **Age (years)** | 24 | 23 | 30 | 24 |
| **Sex** | Male | Male | Female | Male |
| **Leukapheresis time (min)** | 195 | 196 | 237 | 197 |
| **CD34^+^ cells/µl (morning sample)** | 118 | 44 | 20 | 140 |
| **CD34^+^ cells/donorKg** | 12,3 x 10^6^ | 5,5 x 10^6^ | 2,8 x 10^6^ | 11,0 x 10^6^ |
| **Viability** | 98% | 88% | 76% | 96% |


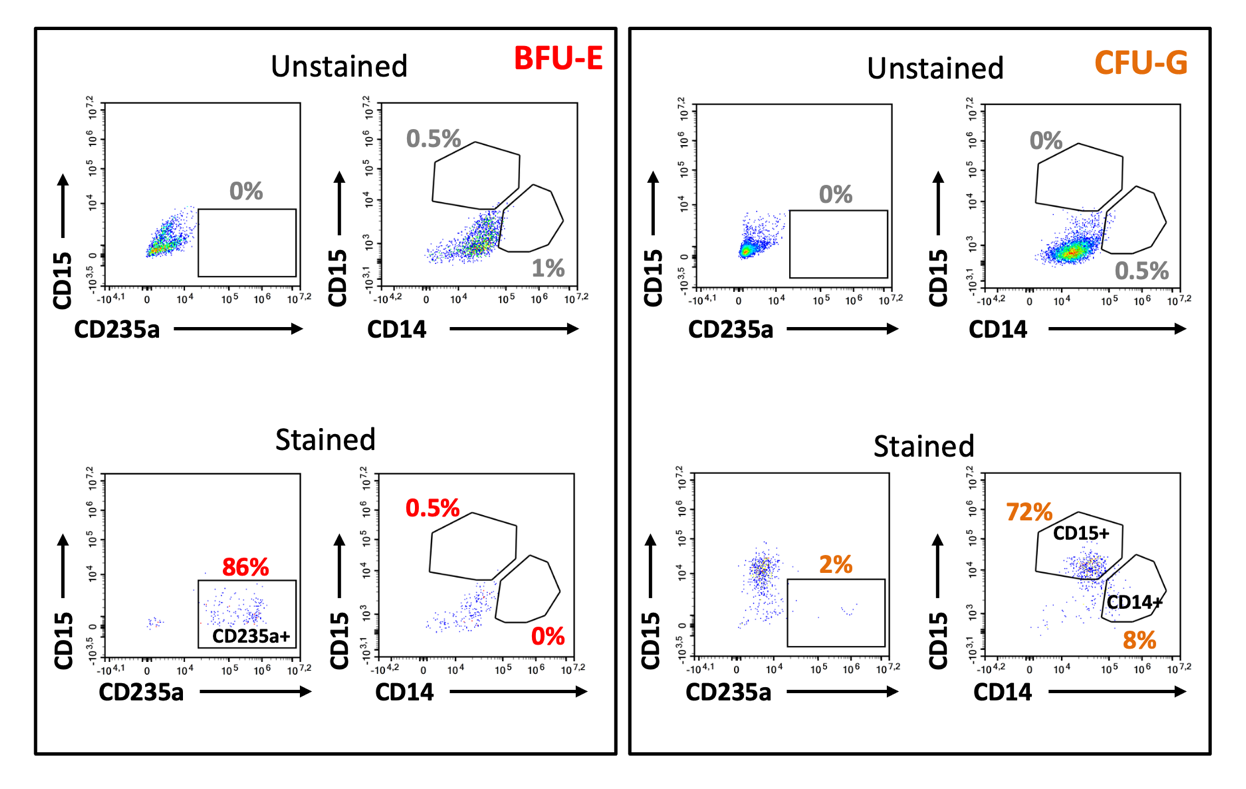


Figure S1. Comparison of unstained negative controls and corresponding stained samples. Flow cytometric acquisition was first performed on five wells from the liquid CFU assay without prior antibody staining. The remaining cells in the same wells were then stained with the assay kit antibody cocktail, and flow cytometric acquisition was repeated. Colonies were identified in three of the five wells; one BFU-E colony and two CFU-G colonies. One representative CFU-G colony is shown. The stained BFU-E colony was used to establish the initial gating strategy, and the resulting gates were subsequently applied to all other samples.

Table S2. Evaluation of intra-assay variation in liquid CFU assay.

| **Gating strategy/Colony assignment** | **%CV** |
| --- | --- |
| FlowJo/Assay limits | 7.15 – 20.09 |
| FlowJo/Recommended limits | 4.38 – 6.02 |
| NovoExpress/Assay limits | 6.14 – 12.97 |
| NovoExpress/Recommended limits | 3.67 - 8.25 |

Intra-assay variation calculated for three independent liquid CFU assay experiments, using CD34+ HSPCs from the same healthy donor. %CV was calculated across the three plates, based on the total number of colonies detected with each combination of gating strategy and colony assignment parameters.

Table S3. Degree of conformity in colony assignment in FlowJo gated data.

| **FlowJo gated** | | **Assay limits** | | | | | | |
| --- | --- | --- | --- | --- | --- | --- | --- | --- |
|  |  | **BFU-E** | **CFU-G** | **CFU-GEMM** | **CFU-GM** | **CFU-M** | **No colony** | **Total** |
| **Recommended** | **BFU-E** | **185** | 0 | 0 | 0 | 0 | 16 | 201 |
|  | **BFU-E & CFU-G** | 42 | 60 | 0 | 0 | 0 | 49 | 151 |
|  | **CFU-G** | 0 | **458** | 0 | 0 | 0 | 56 | 514 |
|  | **CFU-GEMM** | 0 | 0 | **12** | 0 | 0 | 0 | 12 |
|  | **CFU-GM** | 0 | 29 | 0 | **5** | 0 | 14 | 48 |
|  | **CFU-M** | 5 | 0 | 0 | 0 | **12** | 9 | 26 |
|  | **No colony** | 0 | 0 | 0 | 0 | 0 | **200** | 200 |
|  | **Total** | 232 | 547 | 12 | 5 | 12 | 344 | **1152** |

After gating data in the FlowJo software, either Assay limits or Recommended limits were applied as assay detection parameters (Table 1). For each well analysed, (3 plates/donor, 4 healthy donors; 1152 wells in total), the agreement of the resulting colony type was compared. This included double assignments (BFU-E & CFU-G) and empty wells (No colony). The Assay limits produced no double assignments. The highlighted cells in the diagonal represent the number of the particular colony assignments that the two detection parameters agree upon.

Table S4. Degree of conformity in colony assignment in NovoExpress gated data.

| **NovoExpress gated** | | **Assay limits** | | | | | | | |
| --- | --- | --- | --- | --- | --- | --- | --- | --- | --- |
|  |  | **BFU-E** | **BFU-E & CFU-G** | **CFU-G** | **CFU-GEMM** | **CFU-GM** | **CFU-M** | **No colony** | **Total** |
| **Recommended** | **BFU-E** | **136** | 0 | 0 | 0 | 0 | 0 | 15 | 151 |
|  | **BFU-E & CFU-G** | 81 | **6** | 71 | 0 | 0 | 0 | 38 | 196 |
|  | **CFU-G** | 0 | 0 | **449** | 0 | 0 | 0 | 47 | 496 |
|  | **CFU-GEMM** | 0 | 0 | 0 | **6** | 0 | 0 | 0 | 6 |
|  | **CFU-GM** | 0 | 0 | 44 | 0 | **21** | 3 | 12 | 80 |
|  | **CFU-M** | 3 | 0 | 0 | 0 | 0 | **17** | 6 | 26 |
|  | **No colony** | 0 | 0 | 0 | 0 | 0 | 0 | **197** | 197 |
|  | **Total** | 220 | 6 | 564 | 6 | 21 | 20 | 315 | **1152** |

After gating data in the NovoExpress software, either Assay limits or Recommended limits were applied as assay detection parameters (Table 1). For each well analysed, (3 plates/donor, 4 healthy donors; 1152 wells in total), the agreement of the resulting colony type was compared. This included double assignments (BFU-E & CFU-G) and empty wells (No colony). The highlighted cells in the diagonal represent the number of the particular colony assignments that the two detection parameters agree upon.


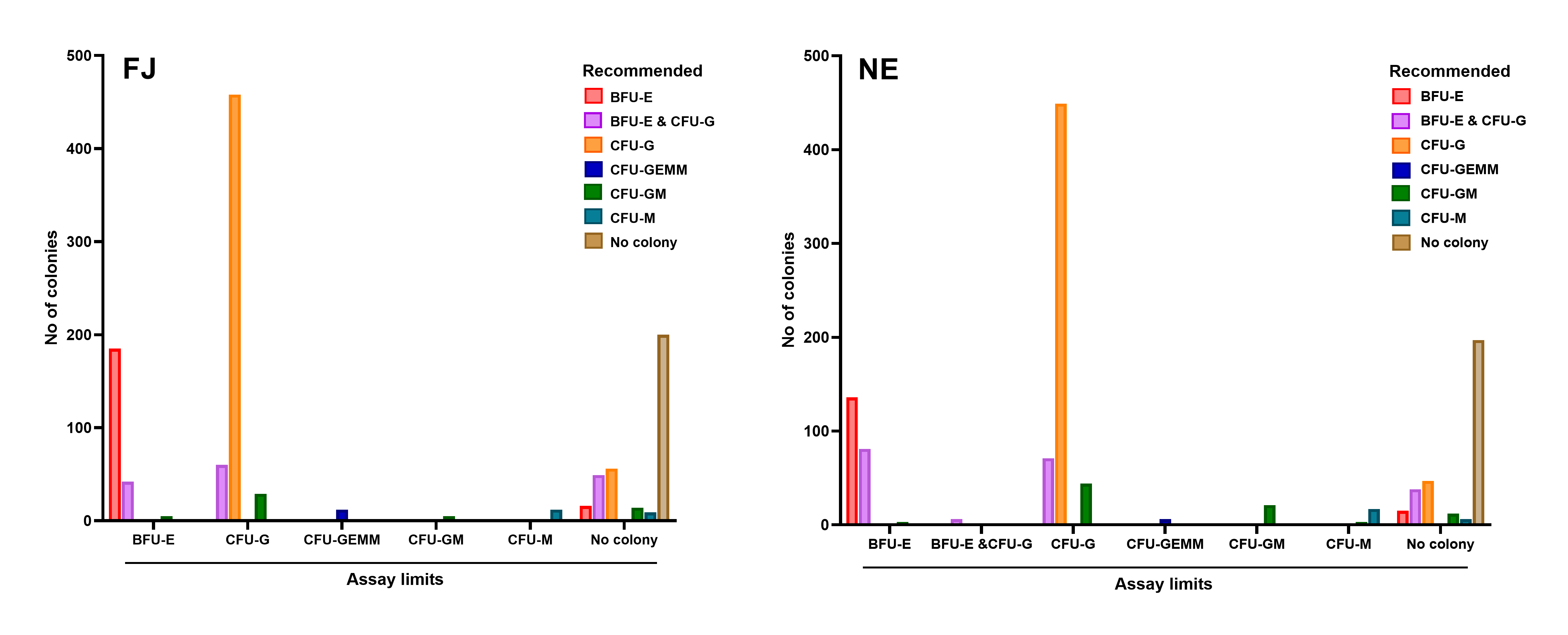


Figure S2. Visualising the level of conformity between different gating strategies and colony assignments. Flow cytometric data acquired from the liquid CFU assay was gated in FlowJo (FJ) or NovoExpress (NE), followed by colony assignment using either assay or recommended limits. The bar plot visualizes the results from the contingency analyses in Supplementary Table 3 and 4. The x axis shows colony assignment based on assay limits, and within each colony type, how the same colonies were assigned using the recommended limits.

Table S5. Evaluation of intra-assay variation in liquid and semi-solid CFU assay.

|  | **%CV**  **Donor 1** | **%CV**  **Donor 2** | **%CV**  **Donor 3** | **%CV**  **Donor 4** |
| --- | --- | --- | --- | --- |
| **Liquid CFU assay** | | | | |
| FlowJo/Assay limits | 3.06 | 0.00 | 11.89 | 11.84 |
| FlowJo/Recommended | 7.79 | 2.47 | 3.86 | 4.38 |
| NovoExpress/Assay limits | 3.51 | 2.57 | 13.15 | 9.12 |
| NovoExpress/Recommended | 8.15 | 2.27 | 1.99 | 2.13 |
| **Semi-solid CFU assay** | | | | |
|  | N/A | 32.39 | 45.29 | 40.32 |

The intra-assay variation was calculated across three 96-well plates (liquid CFU assay) or three discs (semi-solid assay), based on the total number of colonies detected. For the liquid assay, the variation for each combination of gating strategy and colony assignment parameters is included.

Table S6. Cross-assay correlation, using FlowJo as data analysis software.

| **Semi-solid** | **Cross-assay/ Liquid** | | | | | | | |
| --- | --- | --- | --- | --- | --- | --- | --- | --- |
| **Colony type** | **%CD14+** | **%CD15+** | **%CD235a+** | **Assay limits** | **Recommended limits** | **Events CD14+** | **Events CD15+** | **Events CD235a+** |
| **BFU-E** | 1.16 | 9.4 | 85.2 | **BFU-E** | **BFU-E** | 369 | 2990 | 27093 |
| **BFU-E** | 0.39 | 4.95 | 93.6 | **BFU-E** | **BFU-E** | 112 | 1424 | 26905 |
| **BFU-E** | 1.43 | 6.36 | 91 | **BFU-E** | **BFU-E** | 575 | 2549 | 36489 |
| **BFU-E** | 2.08 | 7.93 | 86.9 | **BFU-E** | **BFU-E** | 1096 | 4179 | 45787 |
| **BFU-E** | 2.36 | 8.82 | 85.9 | **BFU-E** | **BFU-E** | 1141 | 4261 | 41519 |
| **CFU-M** | 0 | 0 | 0 | **-** | **-** | 33 | 8 | 11 |
| **CFU-M** | 0 | 0 | 0 | **-** | **-** | 2 | 1 | 3 |
| **CFU-M** | 0 | 0 | 0 | **-** | **-** | 5 | 5 | 6 |
| **CFU-M** | 0 | 0 | 0 | **-** | **-** | 33 | 1 | 9 |
| **CFU-M** | 0 | 0 | 0 | **-** | **-** | 16 | 9 | 3 |
| **CFU-G** | 11 | 43.7 | 0 | **-** | **CFU-GM** | 229 | 907 | 29 |
| **CFU-G** | 6.65 | 48 | 0 | **-** | **CFU-G** | 274 | 1976 | 29 |
| **CFU-G** | 9.22 | 44 | 1.49 | **-** | **CFU-G** | 490 | 2337 | 79 |
| **CFU-G** | 10.1 | 38.6 | 2.01 | **-** | **CFU-GM** | 492 | 1879 | 98 |
| **CFU-G** | 9.05 | 40.1 | 1.45 | **-** | **CFU-G** | 449 | 1992 | 72 |
| **CFU-GEMM** | 2.35 | 12.2 | 78 | **BFU-E** | **BFU-E** | 247 | 1282 | 8200 |
| **CFU-GEMM** | 2.41 | 8.86 | 83.5 | **BFU-E** | **BFU-E** | 457 | 1678 | 15814 |
| **CFU-GEMM** | 1.28 | 15.2 | 72.3 | **BFU-E** | **BFU-E & CFU-G** | 248 | 2954 | 14016 |
| **CFU-GEMM** | 1.1 | 15.4 | 77 | **BFU-E** | **BFU-E & CFU-G** | 285 | 3986 | 19946 |
| **CFU-GEMM** | 1.57 | 20 | 72.8 | **BFU-E** | **BFU-E & CFU-G** | 177 | 2257 | 8226 |
| **CFU-GM** | 0 | 0 | 0 | **-** | **-** | 27 | 2 | 16 |
| **CFU-GM** | 17.4 | 12.2 | 0 | **-** | **-** | 104 | 73 | 34 |
| **CFU-GM** | 19.8 | 20.5 | 5,14 | **-** | **CFU-GM** | 270 | 280 | 70 |
| **CFU-GM** | 0 | 16.9 | 0 | **-** | **-** | 16 | 39 | 9 |
| **CFU-GM** | 0 | 0 | 0 | **-** | **-** | 25 | 9 | 2 |

Five colonies of each type were picked from the semi-solid CFU assay and evaluated with the antibody mix from the liquid CFU assay, allowing evaluation of surface expression of CD14, CD15, and CD235a on the picked colonies. The FlowJo software was applied to perform the gating, after which the colonies were assigned using either the assay or recommended limits (Table 1). The percentwise distribution of each marker is presented along with the number of events detected after the FlowJo gating analysis.

Table S7. Cross-assay correlation, using NovoExpress as data analysis software.

| **Semi-solid** | **Cross-assay/ Liquid** | | | | | | | |
| --- | --- | --- | --- | --- | --- | --- | --- | --- |
| **Colony type** | **%CD14+** | **%CD15+** | **%CD235a+** | **Assay limits** | **Recommended limits** | **Events CD14+** | **Events CD15+** | **Events CD235a+** |
| **BFU-E** | 0 | 2.1 | 94.33 | **BFU-E** | **BFU-E** | 6 | 603 | 27078 |
| **BFU-E** | 0 | 0.99 | 98.39 | **BFU-E** | **BFU-E** | 0 | 269 | 26690 |
| **BFU-E** | 0 | 0.97 | 98.26 | **BFU-E** | **BFU-E** | 2 | 377 | 38021 |
| **BFU-E** | 0 | 1.88 | 95.69 | **BFU-E** | **BFU-E** | 18 | 936 | 47744 |
| **BFU-E** | 0.25 | 2.23 | 96.29 | **BFU-E** | **BFU-E** | 114 | 1017 | 43968 |
| **CFU-M** | 70.77 | 0 | 0 | **CFU-M** | **CFU-M** | 46 | 7 | 1 |
| **CFU-M** | 0 | 0 | 0 | **-** | **-** | 3 | 1 | 0 |
| **CFU-M** | 0 | 0 | 0 | **-** | **-** | 5 | 6 | 3 |
| **CFU-M** | 74.07 | 0 | 0 | **CFU-M** | **CFU-M** | 40 | 6 | 1 |
| **CFU-M** | 34.26 | 40.74 | 0 | **CFU-GM** | **CFU-GM** | 37 | 44 | 0 |
| **CFU-G** | 14.12 | 51.87 | 0 | **CFU-G** | **CFU-GM** | 276 | 1014 | 0 |
| **CFU-G** | 9.38 | 71.5 | 0 | **CFU-G** | **CFU-G** | 370 | 2820 | 1 |
| **CFU-G** | 10.82 | 66.11 | 0 | **CFU-G** | **CFU-GM** | 552 | 3374 | 7 |
| **CFU-G** | 13.28 | 58.75 | 0 | **CFU-G** | **CFU-GM** | 622 | 2752 | 11 |
| **CFU-G** | 12.99 | 67.5 | 0 | **CFU-G** | **CFU-GM** | 622 | 3231 | 8 |
| **CFU-GEMM** | 0.47 | 2.93 | 90.5 | **BFU-E** | **BFU-E** | 45 | 279 | 8609 |
| **CFU-GEMM** | 0 | 1.43 | 93.9 | **BFU-E** | **BFU-E** | 11 | 255 | 16766 |
| **CFU-GEMM** | 0.92 | 4.52 | 82.93 | **BFU-E** | **BFU-E** | 150 | 736 | 13497 |
| **CFU-GEMM** | 0 | 3.3 | 91.54 | **BFU-E** | **BFU-E** | 14 | 718 | 19910 |
| **CFU-GEMM** | 0 | 4.6 | 93.29 | **BFU-E** | **BFU-E** | 6 | 421 | 8544 |
| **CFU-GM** | 0 | 0 | 0 | - | - | 23 | 2 | 1 |
| **CFU-GM** | 33.03 | 46.93 | 0 | **CFU-GM** | **CFU-GM** | 183 | 260 | 7 |
| **CFU-GM** | 30.15 | 59.05 | 0 | **CFU-GM** | **CFU-GM** | 388 | 760 | 14 |
| **CFU-GM** | 0 | 74.09 | 0 | **CFU-G** | **CFU-G** | 19 | 163 | 1 |
| **CFU-GM** | 36.92 | 36.92 | 0 | **CFU-GM** | **CFU-GM** | 48 | 48 | 1 |

Five colonies of each type were picked from the semi-solid CFU assay and evaluated with the antibody mix from the liquid CFU assay, allowing evaluation of surface expression of CD14, CD15, and CD235a on the picked colonies. The NovoExpress software was applied to perform the gating, after which the colonies were assigned using either the assay or recommended limits (Table 1). The percentwise distribution of each marker is presented along with the number of events detected after the NovoExpress gating analysis.


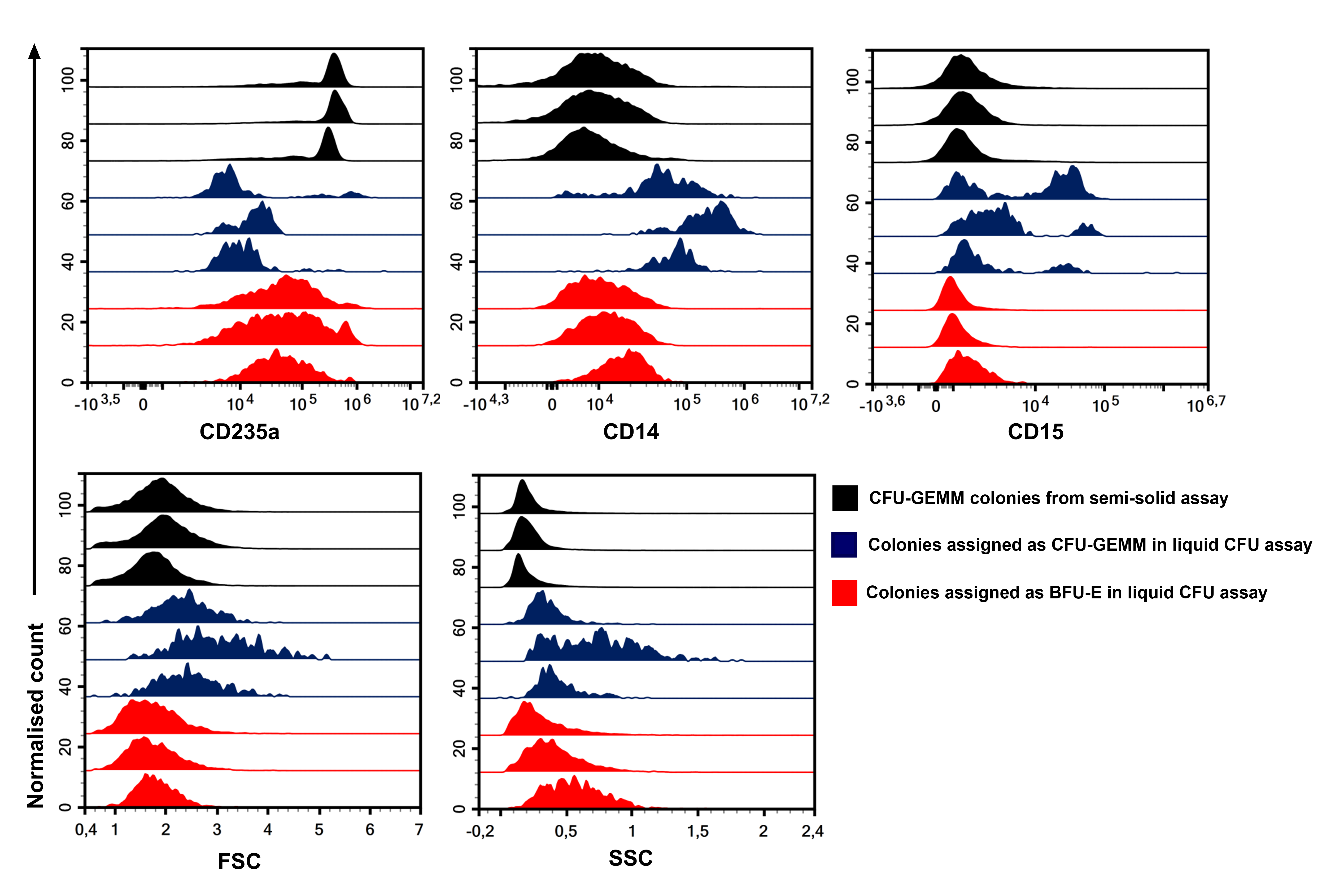


Figure S3. Flow cytometric evaluation of selected colony types from semi-solid and liquid CFU assays. To evaluate cross-assay correlation, colonies identified in the semi-solid assay were examined for their surface expression of CD253a, CD14, and CD15, using the detection platform from the liquid CFU assay. Representative flow cytometric results is presented for three CFU-GEMM colonies picked from the semi-solid assay (black), as well as for three colonies assigned as CFU-GEMM (blue) or BFU-E (red) in the liquid assay, using cells from the same stem cell donor, Donor 4. The histograms show the normalized fluorescence signal for each of the evaluated surface markers, as well as forward (FSC) and side scatter (SSC) parameters.

Table S8. Comparing time consumption and assay costs of liquid CFU assay and semi-solid CFU assay, for the preparation of one sample in technical triplicates.

| **Workflow component** | **Liquid CFU assay** | **Semi-solid CFU assay** | **Notes** |
| --- | --- | --- | --- |
| Format | 96 well plate, round bottom  3 plates/setup  (one conc. of cells) | 35 mm discs/ petri dish  6 discs/ setup  (two conc. of cells) | Multipipette usage in liquid assay |
| Setup (Day 1) | 1,5 h | 3-4 h | Includes media prep., cell count, and plating |
| Incubation | 14 days | 14 days | Typically, identical between assays |
| Instrument time | Acq. time/ plate: 70 min  Total acq. time: 3,5 h  Operator time: 45-60 min | N/A | Flow cytometer required for liquid assay. Operator time includes cell staining |
| Data analysis | 1-1,5 h (batch mode) | 1,5 – 3* h (manual scoring) | Operator-dependent |
| Total hands-on time | 4 h | 4,5 - 7 h |  |
| Estimated cost/run | ~ 27 € | ~ 65 € | Based on local pricing |

The comparison is based on the reagents, applied instruments, and data analysis platforms included in the current study. Hands-on time for liquid assay is assessed as time the operator is required at the instrument. The estimated costs only include the actual reagent usage for the CFU assay (assay kit for liquid CFU assay and media for semi-solid CFU assay). Therefore, additional costs for other consumables, such as pipette tips, plates, and labor cost, will depend on individual laboratory availabilities and infrastructure. For the liquid assay, optimization has shown that 17 mL and not 15 mL (as recommended by the kit manufacturer) is required/ setup. Hence, 26 setups can be made from one kit. For the semi-solid assay, optimization has shown that 80 plates can be made from 100 mL of media. *The data analysis time is dependent on operator experience and therefore a wider time interval is reported.

Table S9. Expression of CD133 by CD34+ HSPC from healthy donors in the study.

| **Donor ID** | **CD34+ CD133+** |
| --- | --- |
| Donor 1 | 84% |
| Donor 2 | 75% |
| Donor 3 | 77% |
| Donor 4 | 85% |

Using flow cytometry, the expression of CD133 was determined for the enriched CD34+ HSPC from all four healthy donors included in the study. After excluding debris, double events, and dead cells, the CD45+ CD34+ events was evaluated for co-expression of CD133, using anti-human CD133-APC (clone W6B3C1, BD Biosciences) as staining reagent.
